# Supplementary material for: Galectin-1 and Galectin-3 in B-Cell Precursor Acute Lymphoblastic Leukemia
Source: Int J Mol Sci. 2022 Nov 18;23(22):14359. doi: 10.3390/ijms232214359 (PMC9694201; doi:10.3390/ijms232214359)
Supplement: Supplementary file 1 [file ijms-23-14359-s001.zip › ijms-1958656-supplementary.pdf]

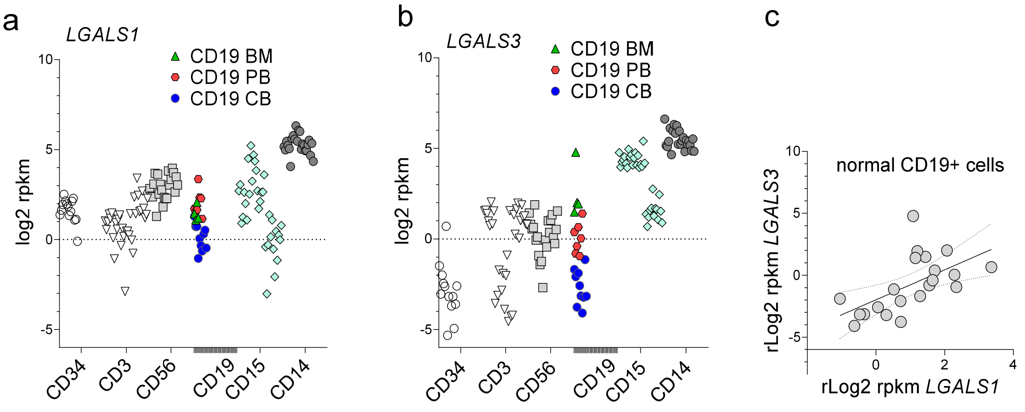

Figure S1. Expression of *LGALS1* and *LGALS3* in normal human hematopoietic cells. (**a, b**) RNA-seq rLog2 values. Each symbol represents one sample. CD19-positive cells are further marked by origin from bone marrow (BM), peripheral blood (PB) or cord blood (CB). CD34, CD3, CD56, CD19, CD15 and CD14 were used as major markers for the following lineages, respectively: hematopoietic stem cell, T cell, NK cell, B cell, myeloid cell, myeloid cell /macrophage (**c**) positive correlation between rLog2 rpkm *LGALS1* and *LGALS3* expression in CD19-positive cells (CB+PB+BM). The dotted line indicates 95% confidence range of best fit line. $R^2 = 0.341$, p = 0.0054. RNAseq data: [1]

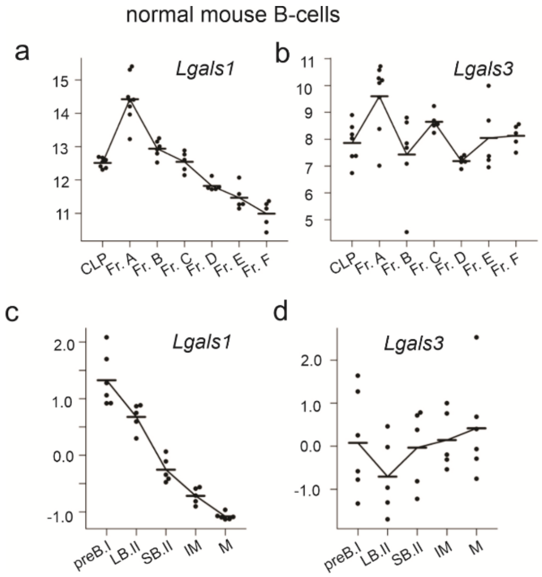

Figure S2. Murine *Lgals1* and *Lgals3* are both expressed during normal B-cell development but in a different pattern. (**a, b**) *Lgals1* and *Lgals3* expression during normal mouse B cell development. Gene expression (GSE38463) of 41 samples from flow-sorted B-cell precursor populations from common lymphoid progenitor (CLP) through to Hardy stage F [2]. Values, MFI of individual samples. (**c, d**) Gene expression (GSE13) of 5 stages of normal B-cell development on flow-sorted or *ex vivo* cells including pre-BI, large pre-BII, small pre-BII, immature B and mature B cell stages. Each dot represents an independent replicate. Normalized average difference values as described [3].

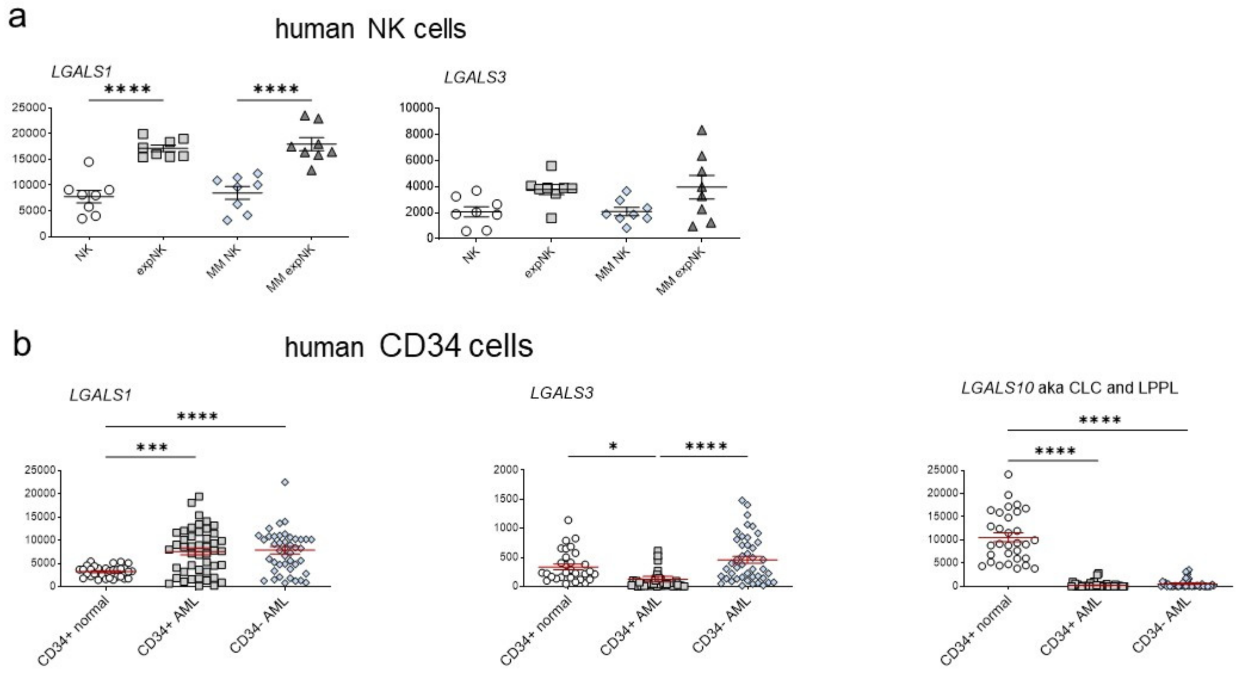

Figure S3. *LGALS1* and *LGALS3* have overlapping expression in normal and abnormal human hematopoietic cells. (**a**) *LGALS1* and *LGALS3* RNA expression in human NK cells (GSE27838) from eight healthy donors and multiple myeloma patients, and NK cells from the same two groups after *ex vivo* expansion in the presence of K562-mb15-41BBL cells [4]. Log-transformed GEP intensity values. Affymetrix genome arrays. *p=0.0228; ***p<0.001; ****p<0.0001. One-way ANOVA, Tukey's multiple comparisons test. (**b**) *LGALS1* and *LGALS3* expression (GSE30029) compared to previously reported *LGALS10* differential expression [5] in AML cells. Magnetic bead-isolated normal bone marrow CD34+ progenitors (n= 31) compared to flow-sorted AML CD34+ and CD34- mononuclear cells [6]. Illumina BeadChip arrays; each symbol, one sample.

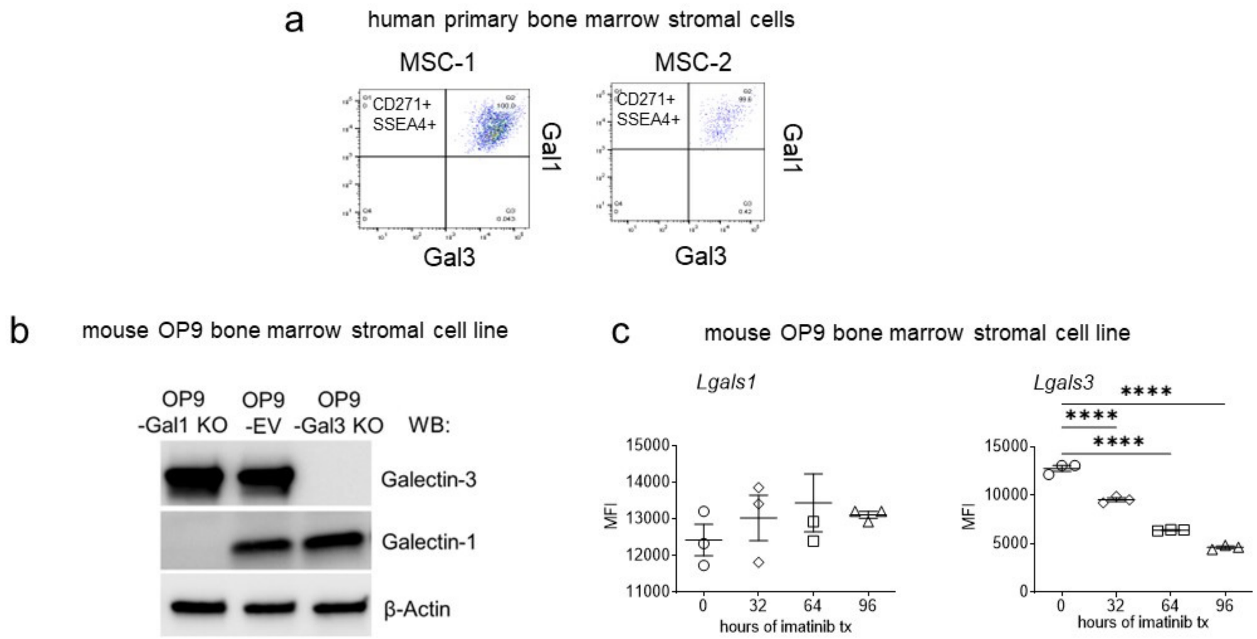

Figure S4. Galectin-1 and Galectin-3 are both expressed in bone marrow stromal cells. (**a**) Normal human bone marrow mesenchymal stromal cells from two individuals described in [7] analyzed by FACS for both Galectin-3 and Galectin-1 expression. (**b**) OP9 stromal cells with Galectin-1 or Galectin-3 knockout. OP9-EV is a control transduced with an empty vector. 4-20% gel. Western blot using antibodies against Galectin-1 (R&D, 1:1000), Galectin-3 (Biolegend, 1:1000, rat) and β-actin-HRP (Santa Cruz, 1:500). (**c**) Expression (GSE56472) of *Lgals1* and *Lgals3* in OP9 bone marrow stromal cells treated with imatinib for the indicated time. Triplicate samples, Illumina GeneChip [8]. Differences between *Lgals1* expression values are not significant. *Lgals3*, significant (****$p<0.0001$) at all time points compared to t = 0. One-way ANOVA, multiple comparisons.

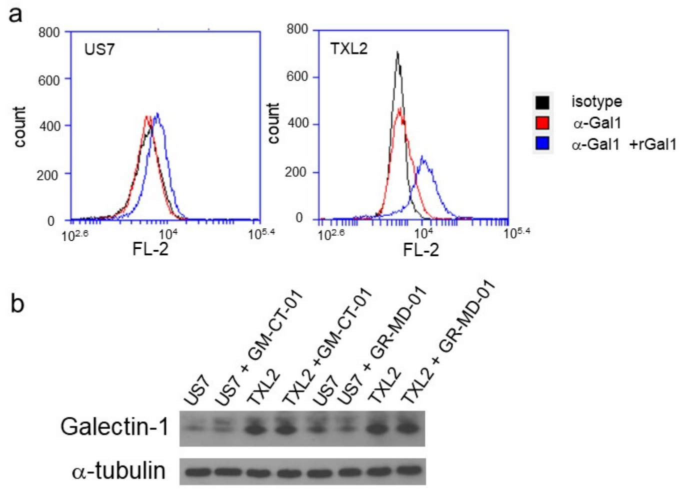

Figure S5. Galectin-1 on BCP-ALL cells. **(a)** The indicated BCP-ALL cells were incubated for 1 hr with 20 μg/ml recombinant human Galectin-1 (rGal1) protein isolated as described [9]. Detection of cell-surface bound Galectin-1 was done on non-fixed, non-permeabilized cells using anti-Galectin-1 antibodies. Note that the signal of Galectin-1 and isotype control antibodies completely or partly overlap, consistent with previous results showing most of the Galectin-1 in these cells is intracellular [9]. (**b**) Western blot analysis for effect of treatment with GM-CT-01 [20 mg/ml] or GR-MD-01 [10 mg/ml] on reducing levels of Galectin-1 on US7 or TXL2 BCP-ALL cells. α-tubulin, loading control.

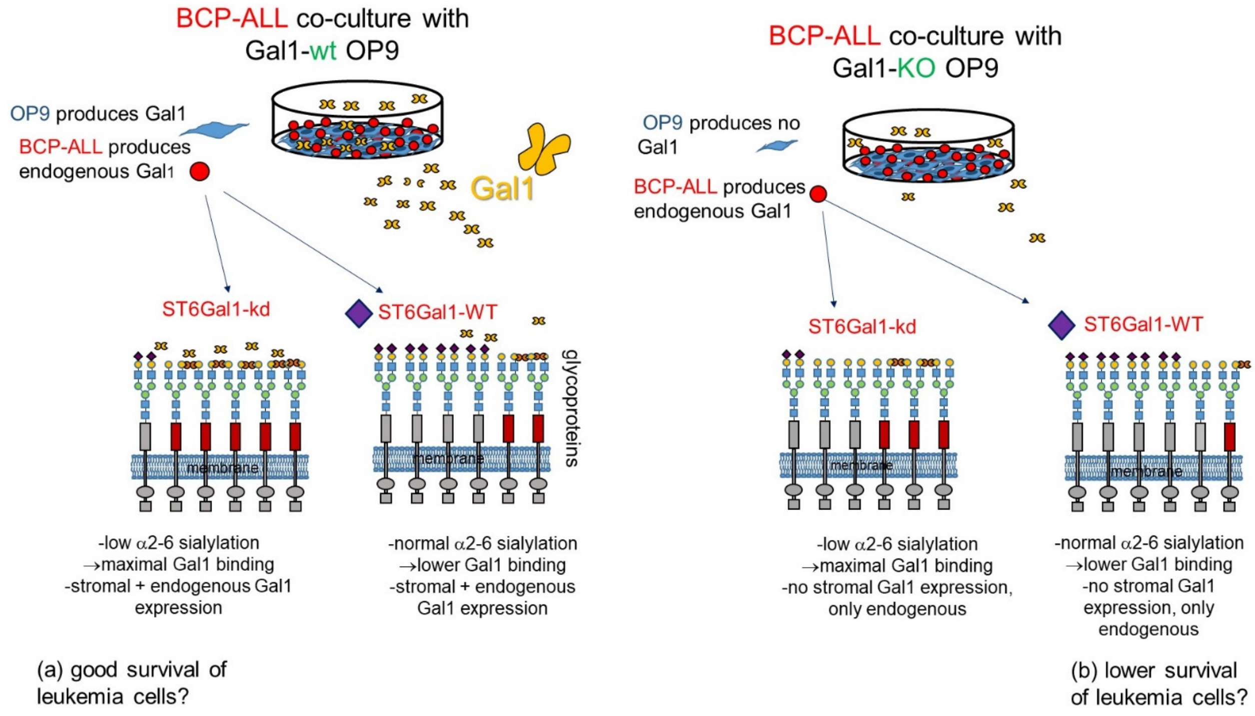

Figure S6. Schematic illustration of the possible effects of ST6Gal1 enzyme activity on the ability of exogenous Galectin-1 to bind to cell surface glycoproteins in BCP-ALL cells. Galectin-1 (and Galectin-3) can bind to both N-glycans and O-glycans containing terminal lactosamine, structures that we recently reported to be both expressed on primary BCP-ALL cells [10]. ST6Gal1 sialylates N-glycans and inhibits Galectin-1 (and some Galectin-3) binding to glycoproteins that carry them [11]. However, sialylation of O-glycans is not affected by ST6Gal1 loss and therefore O-glycans (not illustrated here) can still be bound by Galectin-1 and Galectin-3.
Top panels: two sources of Galectin-1: the leukemia cells (BCP-ALL) and/or the stromal OP9 BM-MSC cells. Galectin-1 is made by BM-MSC and by BCP-ALL cells.
Bottom panels: ST6Gal1 attaches Sia to glycoproteins. This inhibits the ability of Galectin-1 dimers to cross-link target client N-glycoproteins into lattices.
ST6Gal1 enzymatic activity results in loss of binding of Galectin-1 to key N-glycoprotein targets.

Knock down (kd) of ST6Gal1 increases the number of N-glycan binding sites available for Galectin-1 if Galectin-1 is present. As illustrated, quantitative binding of Galectin-1 can regulate signal transduction strength of transmembrane receptors on the plasma membrane by cross-linking them. Depending on the nature of the signals (pro- or anti-survival), this could lead to (a) better or (b) lower ability to resist the stress of vincristine treatment.

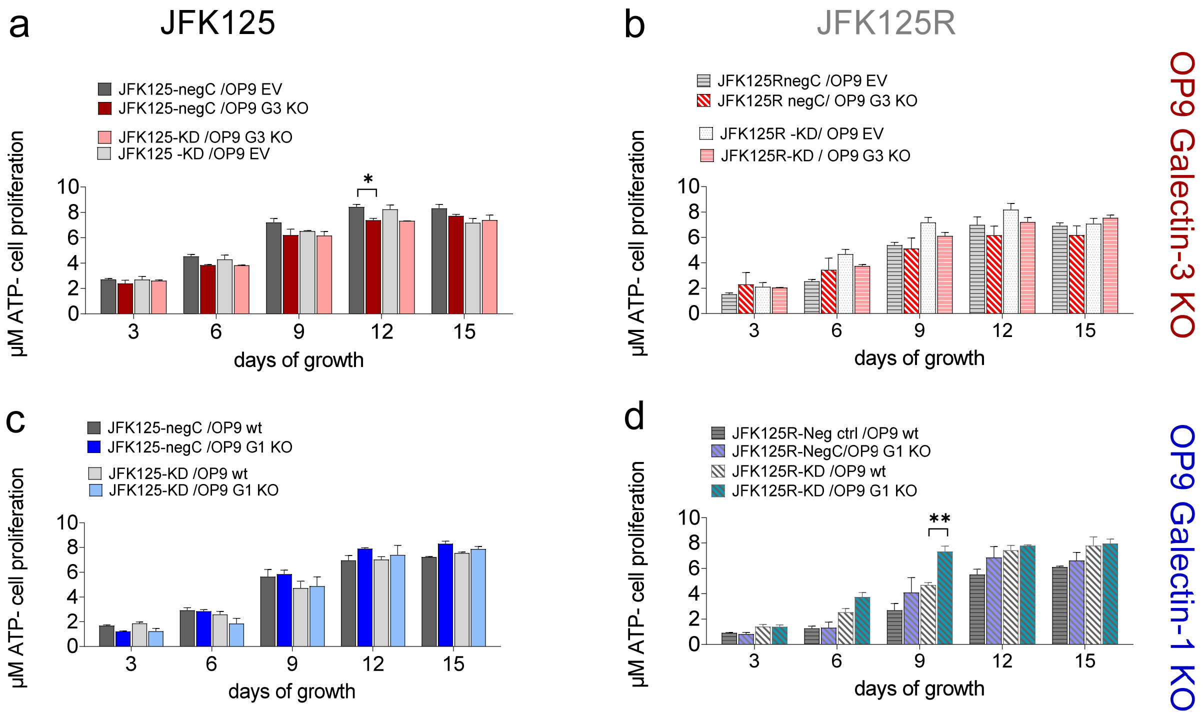

Figure S7. Steady-state growth of the indicated human BCP-ALL leukemia cells on OP9 Galectin-3 and Galectin-1 knockout stromal cells is comparable. ATP levels in BCP-ALL cells in the medium were measured against a standard curve. CellTiterGlo assay, Promega. (**a, c**) JFK125 *ST6GAL1* knockdown or negative control cells or (**b, d**) JFK125R *ST6GAL1* knockdown or negative control cells [12] were co-cultured for 15 days with OP9 Galectin-1 or Galectin-3 knockout cells. **p<0.01, 2-way ANOVA, pair-wise comparisons only between matched BCP-ALLs on OP9 control *versus* OP9 KO cells. n=2 samples per time point. JFK125 and JFK125R are Ph-like subtype BCP-ALL and contain the P2RY8-CRLF2 fusion [13].

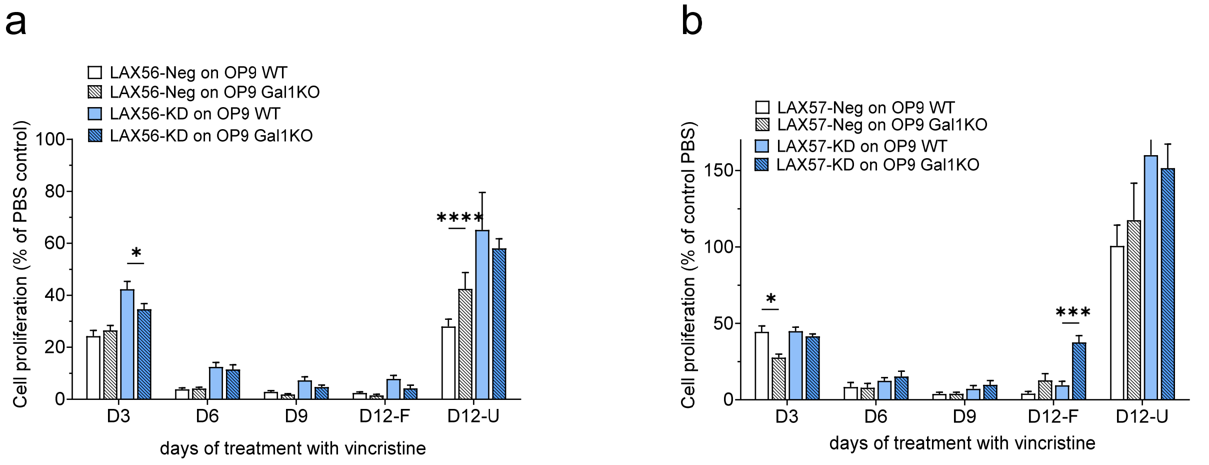

Figure S8. OP9 stromal cells lacking Galectin-1 expression support drug resistance development of LAX56 and LAX57 cells. (**a**) LAX56 cells treated with 7.5 nM vincristine (b) LAX57 cells treated with 4.0 nM vincristine. Cell proliferation by CellTiterGlo assay for ATP. Results are expressed as percentage of the ATP in PBS-treated controls grown in parallel for the same number of days. On day 12, BCP-ALL cells in the supernatant (D12-F) were harvested and analyzed separately from the BCP-ALL cells attached to the top and growing underneath the stromal cell layer (D12-U). *p<0.5; ***p<0.001, two-way ANOVA, multiple pair-wise comparisons between cells grown on OP9 wt and OP9 Galectin-1 knockout stromal cells, adjusted p-value. n = 3-4 samples per time point. Note that BCP-ALL cells associated with the stromal layer overall had higher growth rates compared to BCP-ALL cells that had migrated into the medium.

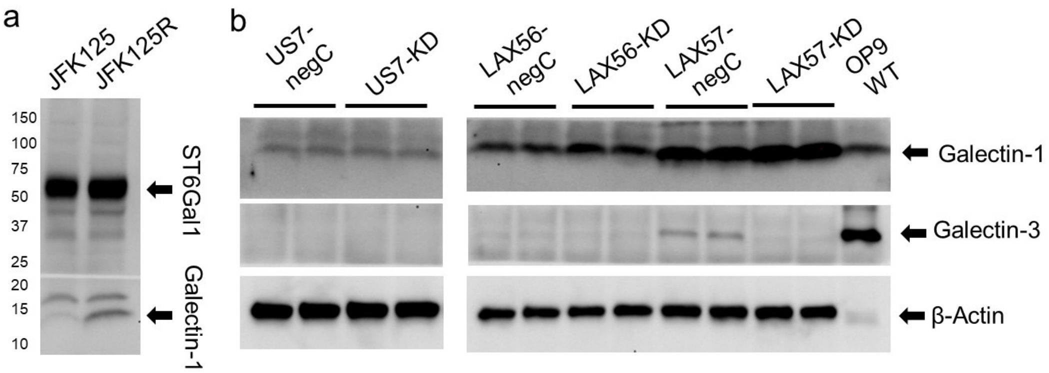

Figure S9. Non-stressed BCP-ALLs express little endogenous Galectin-3 but contain different endogenous levels of Galectin-1. Western blot of the indicated BCP-ALL cells including ST6Gal1 knockdown cells (KD) and negative control wild type cells (negC). Protein concentrations were determined by BCA assay. Antibodies used are as indicated. Samples in ‘b’ are loaded in duplicate with 2x10^{6} cell equivalents/well. Cells in the medium were collected for Western blot analysis. 4-20% SDS-PAA gradient gel.

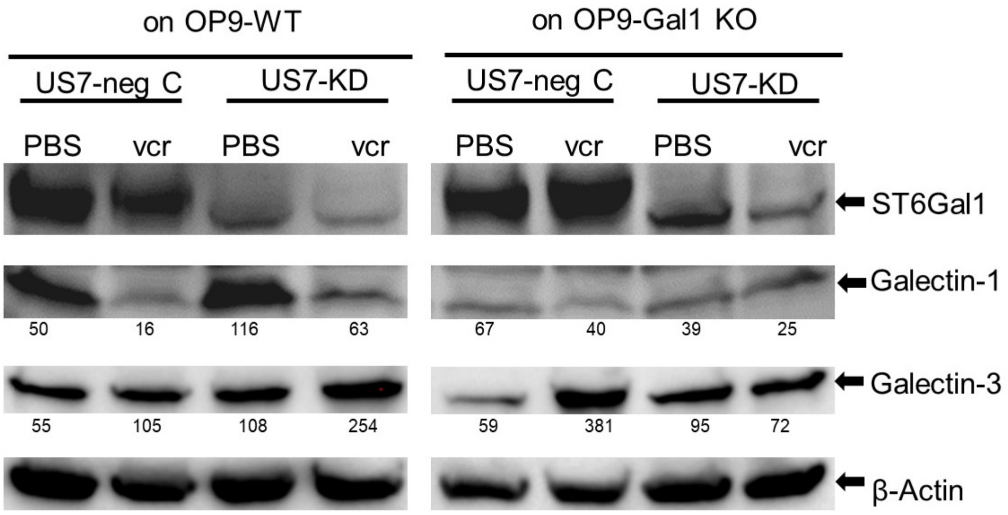

Figure S10. Vincristine treatment does not induce increased Galectin-1 expression in US7 BCP-ALL cells. Cells including those migrated into the medium and attached to the OP9 stroma were harvested on d15 of treatment with vincristine or PBS. Antibodies used are indicated to the right. β-actin, loading control. Numbers, % of signal of β-actin loading control in each lane by Image J quantification of shorter chemiluminescent exposures of the same Western blot.

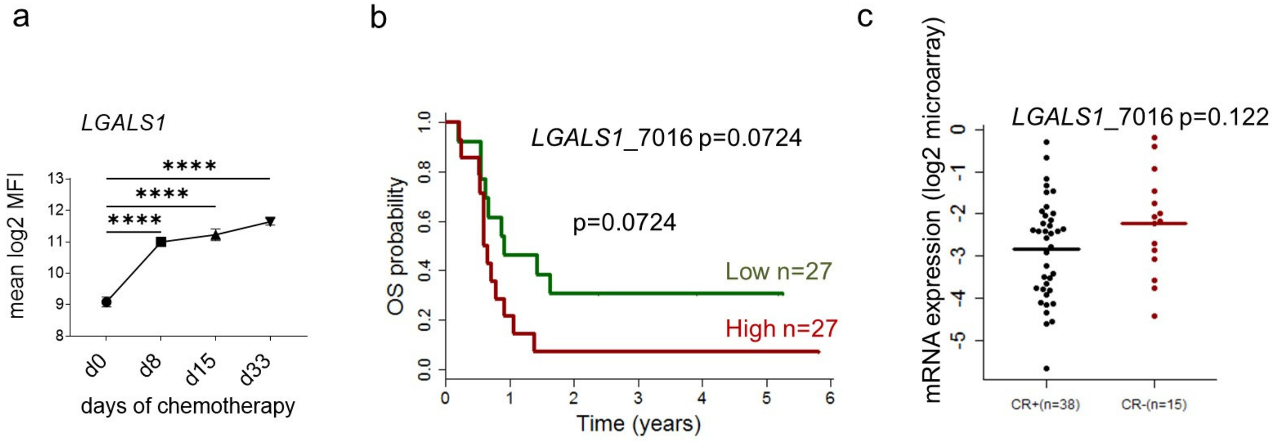

Figure S11. Clinical correlates with *LGALS1* expression in BCP-ALL samples. **(a)** Mean log-transformed normalized GEP values for the indicated genes on 220 pediatric de novo ALL at diagnosis, day 8, day 15, and day 33 of remission-induction therapy (GSE67684). ****p<0.0001, one-way ANOVA, comparisons with d0. (**b, c**) Adult ALL ECOG E2993; GSE5314. (**b**) n=54 samples. Median value of *LGALS1* expression was determined for all samples. Based on the median value, samples were grouped into those with higher/equal or lower than the median value. OS probability is lower for patients with high average *LGALS1*. p=0.0724. (**c**) Patients who achieved a complete remission (CR^{pos}, n=38) compared those who did not (CR^{neg}, n=15). Average values, p = 0.122, ns; logrank test.

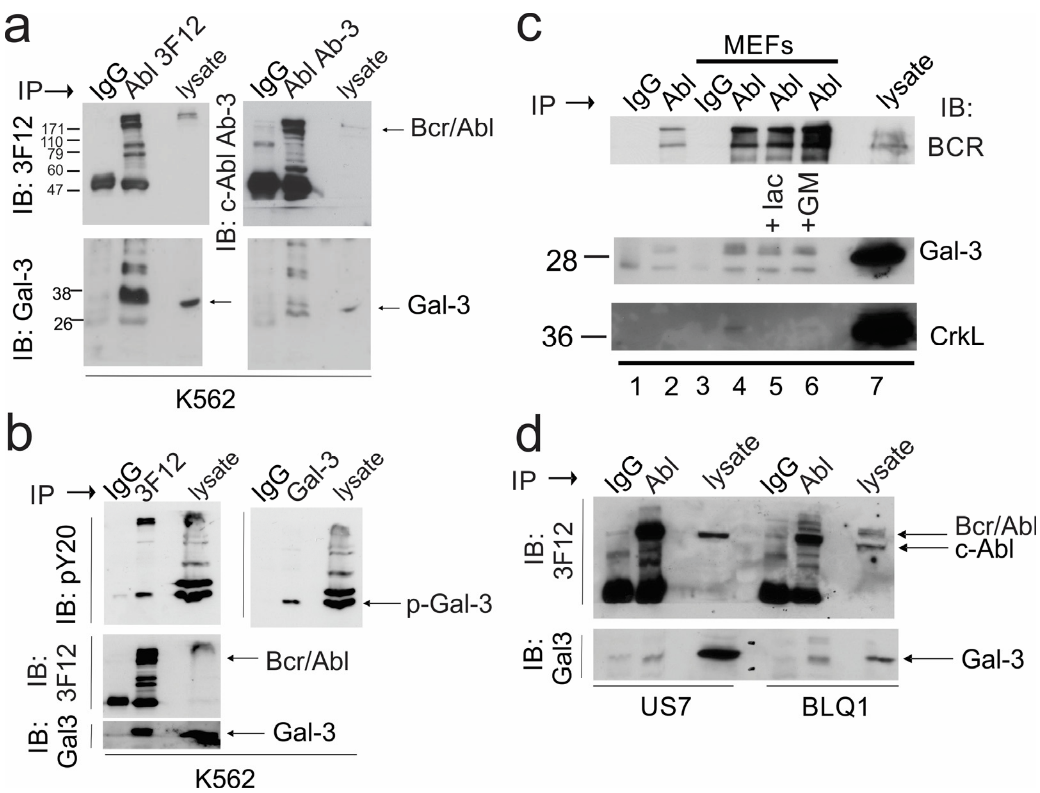

Figure S12. Complex formation of Galectin-3 with (Bcr/)Abl in leukemia cells. Lysates made from (**a-c**) the CML cell line K562 or (**d**) BCP-ALLs. **(a).** Immunoprecipitation with control Ig or two different anti-c-Abl antibodies: 3F12 or c-Abl Ab-3 (Calbiochem) as indicated above the lanes. Antibodies used for immunoblotting are indicated to the left. Arrows to the right of the panels point to the location of Bcr/Abl and Galectin-3 (Gal-3). Note: the right upper panel was exposed longer than the left upper panel. (**b**). Immunoprecipitation with anti-Abl 3F12 or Galectin-3 antibodies followed by immunoblotting with the antibodies indicated to the left of the panels. (**c**) Immunoprecipitation from K562 lysates of cells grown as suspension culture (lanes 1-2) or on MEFs (lanes 3-6) as indicated above the panel. Lane 7, total cell lysate. Antibodies used for IP include control IgG (lanes 1 and 3) or 3F12 anti-Abl (lane 2, lanes 4-6). Lane 5, cells treated for 2 hours with 50 mM lactose; lane 6, cells treated with 10 mg/ml GM-CT-01 for 2 hours. After immunoblotting with Galectin-3, the panel was stripped and re-probed with anti-Crkl antibodies. A 12% SDS-PAA gel was used to obtain separation in the lower molecular weight range. (**d**) Immunoprecipitation of c-Abl and Bcr/Abl from lysates of human BCP-ALL US7 (Ph-negative) and BLQ1 (Ph-positive). Cells were grown on OP9 stroma before preparation of lysates.

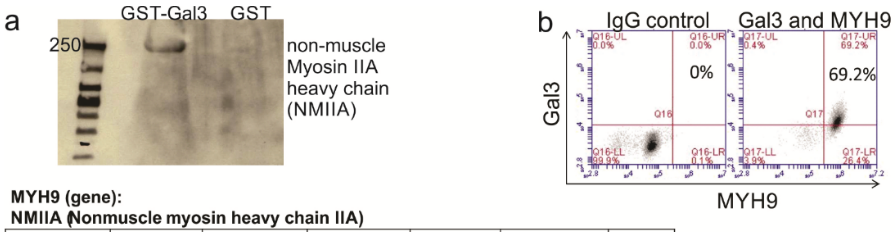

MYH9 (gene):
NMIIA (Nonmuscle myosin heavy chain IIA)

Accession	Score	Coverage	# Proteins	# Unique Peptides	# Peptides	# PSMs
P35579	354.36	38.67	17	56	64	116

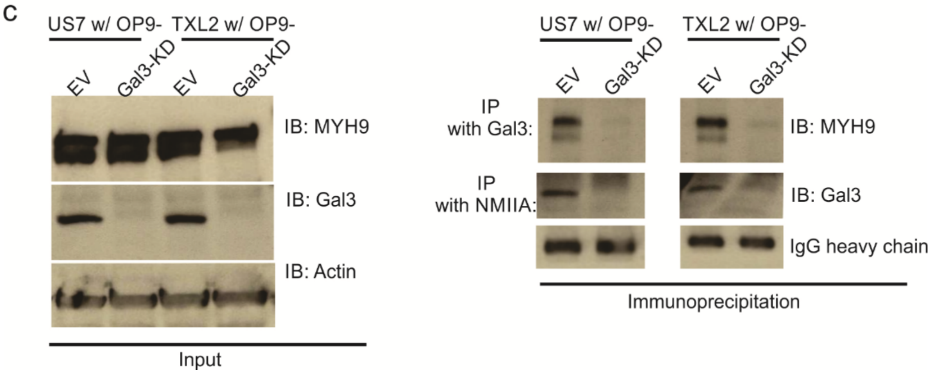

Figure S13. Non-muscle myosin IIA (MYH9, NMIIA) forms a protein complex with Galectin-3 (Gal3) in BCP-ALL cells. (**a**) Western blot detection of MYH9 pulled down with recombinant Galectin-3 in US7 BCP-ALL whole cell lysate. The table shows proteomics data of the excised Coomassie Blue stained band of ~250 kDa. (**b**) FACS analysis of TXL2 cells (in co-culture with OP9 stroma) fixed, permeabilized and double-stained with anti-Galectin-3 and MYH9. (**c**) Left: Western blotting of the indicated BCP-ALLs after 14 days of co-culture with OP9-CRISPR-Galectin-3 knockdown [7] and control OP9. Right: co-IP with anti-Galectin-3 and anti-MYH9 antibodies followed by immunoblotting to detect Galectin-3 and MYH9 in co-IP. Actin, loading control for lysates; heavy chain IgG (50 kDa), loading control for IP.

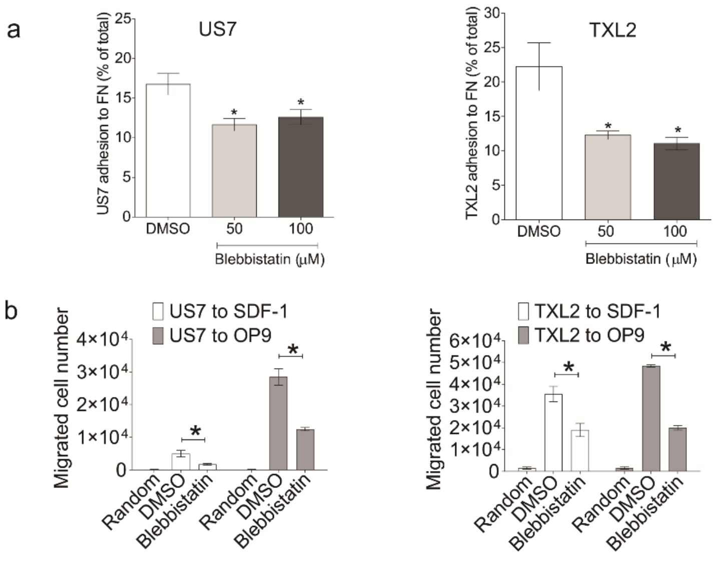

Figure S14. MYH9 inhibition decreases BCP-ALL cell migration and adhesion *in vitro*. (**a**) Adhesion of BCP-ALL cells to fibronectin (FN) in the presence of blebbistatin as specific MYH9 inhibitor. (**b**) Migration of BCP-ALL cells toward stroma (12 hr assay) and 200 ng/ml SDF1-α (2 hr assay) in the presence of 100 µM blebbistatin. Duplicate samples per BCP-ALL, t-test, * $p < 0.05$. Blebbistatin (Sigma-Aldrich, St. Louis, MO) was dissolved in DMSO and stored at -20°C.

Supplementary methods-

Abl co-IP: Anti c-Abl Ab-3 antibodies for immunoprecipitation were from Calbiochem (San Diego CA). For immunoprecipitations, 1 mg of protein was used. Lysates were pre-cleared by incubation with 3 µg rabbit or mouse IgG and PAA. ALL cells were lysed for 30 minutes on ice in RIPA buffer (50 mM Tris-HCl, pH 8.0, 150 mM NaCl, 1% Triton X-100, 0.5% deoxycholate, 0.1% SDS, 5 mM EDTA) containing PMSF, aprotinin, leupeptin, pepstatin A, Na-fluoride and Na-orthovanadate. BLQ1 has been described previously [14] and, similar to TXL2 and K562, expresses the P210 version of Bcr/Abl.

GST-Galectin-3 interactions: For biotin-streptavidin pull down, purified recombinant Galectin-3-GST and GST control (1 mg) 24 were biotinylated (#21335, Thermo Scientific) for 2 hr at 4°C while rotating. After removal of excess biotin (3K spin column, #OD003C33, Pall, Port Washington, NY), the biotinylated proteins were incubated with whole cell lysates (RIPA buffer) isolated from pre-B ALL cells, at 4°C overnight. Streptavidin-conjugated magnetic beads (#65801D, Invitrogen) at 2-fold excess were used to pull-down biotin-bound peptides along with Galectin-3 binding partners (following the manufacturer's instructions). The pull-down product was run on SDS-PAGE gels and stained with Coomassie Blue. A band at ~250 kDa was excised and analyzed by Mass Spectroscopy (University of Southern California Proteomics Core Facility).

NMIIA/MYH9 co-IP: Whole cell lysates for IP were prepared with IP lysis buffer containing 25 mM Tris-HCl pH 7.4, 150 mM NaCl, 1 mM EDTA, 1% NP-40, 0.25% deoxycholic acid (sodium deoxycholate) and 1x protease and phosphatase inhibitors (Roche, Basel, Switzerland). At least 500 µg lysate was used to pull down protein complexes using protein G Agarose beads (#20398, Pierce, Thermo Scientific) and ~4 µg antibodies against Galectin-3 and MYH9/NMIIA whereas 1 µg normal IgG was used as negative control (1:100 ratio). Denatured pull down samples were subjected to regular Western blotting with exception of using TrueBlot IP secondary antibody (Rockland Immunochemicals, Limerick, PA) for immunodetection.

BCP-ALL growth on OP9 Galectin-1 knockout cells: OP9 Galectin-1 and Galectin-3 knockout cells have been previously described [7, 12]. LAX56 and LAX57 were described in [15].

Supplementary results- ABL:

Actin cytoskeletal reorganization is essential for cell migration, and in some cell types Abl was shown to play a critical role in regulating actin dynamics [16]. In leukemias with a BCR/ABL translocation, the actin binding domain of Abl is retained in the fusion protein and the Bcr/Abl protein associates with the actin cytoskeleton via the Abl actin-binding domain. Thus Bcr/Abl also regulates cell migration [17, 18]. Interestingly, Galectin-3 was shown to form a protein complex with Abl in prostate and breast cancer cell lines and becomes phosphorylated on tyrosine [19-21]. However, BioID analysis to characterize Bcr/Abl-interacting proteins did not report complexes with, or tyrosine phosphorylation of, Galectin-3 in mouse hematopoietic cells [22] and one of the most comprehensive analysis of the Galectin-3 interactome reported to date does not include c-Abl [23].

To determine if Bcr/Abl and Galectin-3 interact in leukemia cells that express endogenous Galectin-3, we made use of the CML cell line K562, because it contains multiple copies of the *BCR/ABL* gene [24]. As shown in Figure S12a (lane total lysate, immunoblotted with Galectin-3 antibodies), we confirmed that K562 cells express Galectin-3 protein endogenously in the absence of stromal cells. Interestingly, Galectin-3 was detected in immunoprecipitates with Bcr/Abl using two different anti-c-Abl monoclonal antibodies (Figure S12a, IP Abl 3F12 or IP Abl Ab-3; bottom panel, Gal3 WB). To determine if Galectin-3 becomes tyrosine phosphorylated in these cells, we immunoprecipitated Galectin-3 and used anti-PY20 antibodies to examine the precipitated protein. Figure S12b (right panel: IP Gal3, IB pY20) shows that Galectin-3 is tyrosine phosphorylated and confirmed it is detected in the Bcr/Abl immunoprecipitate (left panel: IP 3F12, IB: Gal3).

To investigate if exogenous Galectin-3 provided by stroma also interacts with Abl, we co-cultured the K562 with MEFs, prepared lysates, and preformed immunoprecipitations with anti-Abl

3F12 antibodies. As shown in Figure S12c, compared to K562 cultured alone, the co-cultured cells contained a higher molecular Galectin-3 weight band (compare lanes 2 and 4, Gal-3 WB) representing murine Galectin-3. Mouse Galectin-3 as well as endogenous human Galectin-3 co-immunoprecipitated with Bcr/Abl (Figure S12c, double band in IPs from K562+MEF lysates, 3F12 IPs, Galectin-3 WB). Re-probing of the membrane with anti-Crkl antibodies showed that Crkl had also been co-immunoprecipitated with Bcr/Abl as expected [25]. These results suggest that both endogenously produced as well as exogenous Galectin-3 bound to or taken up by K562, can interact intracellularly with (Bcr)/Abl.

To confirm that Bcr/Abl indeed forms a complex with exogenously produced Galectin-3, we immunoprecipitated Bcr/Abl from human Ph-positive BLQ1 BCP-ALL cells. These do not express much Galectin-3 endogenously under steady state conditions [26]. Figure S12d (IP 3F12 IB: Galectin-3) shows that we also detected co-immunoprecipitation of Bcr/Abl with Galectin-3 in BLQ1 cells, indicating that mouse Galectin-3 and human Bcr/Abl also interact. As we also detected co-immunoprecipitation of c-Abl with Galectin-3 in US7 cells, which are Ph-chromosome negative and do not contain Bcr/Abl (Figure S12d), interaction of Abl with Galectin-3 may be a common mechanism that could regulate migration of BCP-ALL cells.

Supplementary results- MYH9:

We used proteomics to identify other intracellular Galectin-3-interacting proteins. Biotinylated recombinant Galectin-3 was incubated with whole cell lysates from ALL cells and magnetic beads conjugated to streptavidin were used to pull down Galectin-3 and proteins bound to it. This method identified non-muscle myosin heavy chain IIA (NMIIA) as a major protein interacting with Galectin-3 (Figure S13a). Interestingly, MYH9/NMIIA is an essential component of the cell motility machinery [27], regulating actin polymerization and cell contractility [28].

FACS double staining for both Galectin-3 and MYH9 in BCP-ALL cells in co-culture with OP9 stroma showed co-expression in a high percentage of BCP-ALL cells (Figure S13b), whereas co-immunoprecipitation (co-IP) with antibodies specific for Galectin-3 and MYH9 was used to confirm their endogenous protein-protein interaction in BCP-ALL cells (Figure S13b). We performed the co-IPs in whole cell lysates extracted from BCP-ALL cells expanded on OP9-Galectin-3-CRISPR knockdown stromal cells (described in [7]) and compared the outcome to BCP-ALL cells co-cultured with control OP9-EV. As depicted in Figure S13c, right panel, whereas Galectin-3 and MYH9 found in a co-IP in BCP-ALL cells cultured with normal OP9, no MYH9 was present in an IP with anti-Galectin-3 antibodies using lysates of BCP-ALL cells grown with OP9-Galectin-3-KD. These results were repeated with an IP using anti-MYH9 antibodies (NMIIA, Figure S13c), providing further support for the specificity of affinity between Galectin-3 and MYH9. As the co-immunoprecipitation could not be inhibited by lactose (results not shown) the interaction between these two proteins is not mediated by the Galectin-3 carbohydrate recognition domain binding to glycan modified MYH9. Based on these results, we conclude that Galectin-3 and MYH9 interact directly inside BCP-ALL cells in a carbohydrate-independent manner. These results agree with Joeh et al [23], who also found a lactose-independent interaction between Galectin-3 and MYH9 in peripheral blood mononuclear cells.

To test the direct involvement of MYH9 in regulating BCP-ALL cell adhesion and migration we measured the effect of blebbistatin, a highly specific inhibitor of MYH9 ATPase activity, on these BCP-ALL functions. We selected an effective but non-toxic (>88% cell viability after 48 hr, not shown) dose of blebbistatin (final concentration of 50-100 μM) for MYH9 inhibition in BCP-ALL cells. As shown in Figure S14a, MYH9 inhibition resulted in a significant reduction in BCP-ALL adhesion to fibronectin. We also evaluated the effect of blebbistatin on BCP-ALL cell migration toward SDF1-α (200 ng/ml) and OP9 stromal cells. Incubation of BCP-ALL cells with blebbistatin (100 μM) significantly reduced the extent at which BCP-ALL cells migrated to both SDF-1α and OP9 cells (Figure S14b). These results together suggest that Galectin-3 may modulate the adhesion and migration of BCP-ALL cells through MYH9.

Literature Cited

1. Gu Z, Churchman ML, Roberts KG, Moore I, Zhou X, Nakitandwe J, Hagiwara K, Pelletier S, Gingras S, Berns H, Payne-Turner D, Hill A, Iacobucci I, Shi L, Pounds S, Cheng C, Pei D, Qu C, Newman S, Devidas M, Dai Y, Reshmi SC, Gastier-Foster J, Raetz EA, Borowitz MJ, Wood BL, Carroll WL, Zweidler-McKay PA, Rabin KR, Mattano LA, Maloney KW, Rambaldi A, Spinelli O, Radich JP, Minden MD, Rowe JM, Luger S, Litzow MR, Tallman MS, Racevskis J, Zhang Y, Bhatia R, Kohlschmidt J, Mrozek K, Bloomfield CD, Stock W, Kornblau S, Kantarjian HM, Konopleva M, Evans WE, Jeha S, Pui CH, Yang J, Paietta E, Downing JR, Relling MV, Zhang J, Loh ML, Hunger SP, Mullighan CG. PAX5-driven subtypes of B-progenitor acute lymphoblastic leukemia. Nat Genet. 2019;51(2):296-307. **30643249**.
2. Holmfeldt L, Wei L, Diaz-Flores E, Walsh M, Zhang J, Ding L, Payne-Turner D, Churchman M, Andersson A, Chen SC, McCastlain K, Becksfort J, Ma J, Wu G, Patel SN, Heatley SL, Phillips LA, Song G, Easton J, Parker M, Chen X, Rusch M, Boggs K, Vadodaria B, Hedlund E, Drenberg C, Baker S, Pei D, Cheng C, Huether R, Lu C, Fulton RS, Fulton LL, Tabib Y, Dooling DJ, Ochoa K, Minden M, Lewis ID, To LB, Marlton P, Roberts AW, Raca G, Stock W, Neale G, Drexler HG, Dickins RA, Ellison DW, Shurtleff SA, Pui CH, Ribeiro RC, Devidas M, Carroll AJ, Heerema NA, Wood B, Borowitz MJ, Gastier-Foster JM, Raimondi SC, Mardis ER, Wilson RK, Downing JR, Hunger SP, Loh ML, Mullighan CG. The genomic landscape of hypodiploid acute lymphoblastic leukemia. Nat Genet. 2013;45(3):242-252. **23334668**.
3. Hoffmann R, Seidl T, Neeb M, Rolink A, Melchers F. Changes in gene expression profiles in developing B cells of murine bone marrow. Genome Res. 2002;12(1):98-111. **11779835**.
4. Garg TK, Szmania SM, Khan JA, Hoering A, Malbrough PA, Moreno-Bost A, Greenway AD, Lingo JD, Li X, Yaccoby S, Suva LJ, Storrie B, Tricot G, Campana D, Shaughnessy JD, Jr., Nair BP, Bellamy WT, Epstein J, Barlogie B, van Rhee F. Highly activated and expanded natural killer cells for multiple myeloma immunotherapy. Haematologica. 2012;97(9):1348-1356. **22419581**.
5. Larramendy ML, Niini T, Elonen E, Nagy B, Ollila J, Vihinen M, Knuutila S. Overexpression of translocation-associated fusion genes of FGFRI, MYC, NPMI, and DEK, but absence of the translocations in acute myeloid leukemia. A microarray analysis. Haematologica. 2002;87(6):569-577. **12031912**.
6. de Jonge HJ, Woolthuis CM, Vos AZ, Mulder A, van den Berg E, Kluin PM, van der Weide K, de Bont ES, Huls G, Vellenga E, Schuringa JJ. Gene expression profiling in the leukemic stem cell-enriched CD34+ fraction identifies target genes that predict prognosis in normal karyotype AML. Leukemia. 2011;25(12):1825-1833. **21760593**.
7. Tarighat SS, Fei F, Joo EJ, Abdel-Azim H, Yang L, Geng HM, Bum-Erdene K, Grice ID, von Itzstein M, Blanchard H, Heisterkamp N. Overcoming Microenvironment-Mediated Chemoprotection through Stromal Galectin-3 Inhibition in Acute Lymphoblastic Leukemia. International Journal of Molecular Sciences. 2021;22(22):12167. **34830047**.
8. Mallampati S, Leng X, Ma H, Zeng J, Li J, Wang H, Lin K, Lu Y, Yang Y, Sun B, Gong Y, Lee JS, Konopleva M, Andreeff M, Arlinghaus RB, Cai Z, Fang B, Shen H, Han X, Hirsch-Ginsberg CF, Gao X, Paranjape AN, Mani SA, Clise-Dwyer K, Sun X. Tyrosine kinase inhibitors induce mesenchymal stem cell-mediated resistance in BCR-ABL+ acute lymphoblastic leukemia. Blood. 2015;125(19):2968-2973. **25712988**.
9. Paz H, Joo EJ, Chou CH, Fei F, Mayo KH, Abdel-Azim H, Ghazarian H, Groffen J, Heisterkamp N. Treatment of B-cell precursor acute lymphoblastic leukemia with the Galectin-1 inhibitor PTX008. J Exp Clin Cancer Res. 2018;37(1):67. **29580262**.

10. Oliveira T, Zhang M, Joo EJ, Abdel-Azim H, Chen CW, Yang L, Chou CH, Qin X, Chen J, Alagesan K, Almeida A, Jacob F, Packer NH, von Itzstein M, Heisterkamp N, Kolarich D. Glycoproteome remodeling in MLL-rearranged B-cell precursor acute lymphoblastic leukemia. Theranostics. 2021;11(19):9519-9537. **34646384**.

11. Nielsen MI, Stegmayr J, Grant OC, Yang Z, Nilsson UJ, Boos I, Carlsson MC, Woods RJ, Unverzagt C, Leffler H, Wandall HH. Galectin binding to cells and glycoproteins with genetically modified glycosylation reveals galectin-glycan specificities in a natural context. J Biol Chem. 2018;293(52):20249-20262. **30385505**.

12. Zhang M QT, Yang L, Kolarich D and Heisterkamp, N. . ST6Gal1 expression in precursor B-lineage acute lymphoblastic leukemia. Frontiers in Oncology. 2022.

13. Chan LN, Murakami MA, Robinson ME, Caeser R, Sadras T, Lee J, Cosgun KN, Kume K, Khairnar V, Xiao G, Ahmed MA, Aghania E, Deb G, Hurtz C, Shojaee S, Hong C, Polonen P, Nix MA, Chen Z, Chen CW, Chen J, Vogt A, Heinaniemi M, Lohi O, Wiita AP, Izraeli S, Geng H, Weinstock DM, Muschen M. Signalling input from divergent pathways subverts B cell transformation. Nature. 2020;583(7818):845-851. **32699415**.

14. Fei F, Lim M, Schmidhuber S, Moll J, Groffen J, Heisterkamp N. Treatment of human pre-B acute lymphoblastic leukemia with the Aurora kinase inhibitor PHA-739358 (Danusertib). Mol Cancer. 2012;11:42. **22721004**.

15. George AA, Paz H, Fei F, Kirzner J, Kim YM, Heisterkamp N, Abdel-Azim H. Phosphoflow-Based Evaluation of Mek Inhibitors as Small-Molecule Therapeutics for B-Cell Precursor Acute Lymphoblastic Leukemia. PLoS One. 2015;10(9):e0137917. **26360058**.

16. Tang DD, Gerlach BD. The roles and regulation of the actin cytoskeleton, intermediate filaments and microtubules in smooth muscle cell migration. Respir Res. 2017;18(1):54. **28390425**.

17. Preisinger C, Kolch W. The Bcr-Abl kinase regulates the actin cytoskeleton via a GADS/Slp-76/Nck1 adaptor protein pathway. Cell Signal. 2010;22(5):848-856. **20079431**.

18. Salgia R, Quackenbush E, Lin J, Souchkova N, Sattler M, Ewaniuk DS, Klucher KM, Daley GQ, Kraeft SK, Sackstein R, Alyea EP, von Andrian UH, Chen LB, Gutierrez-Ramos JC, Pendergast AM, Griffin JD. The BCR/ABL oncogene alters the chemotactic response to stromal-derived factor-1alpha. Blood. 1999;94(12):4233-4246. **10590068**.

19. Balan V, Nangia-Makker P, Kho DH, Wang Y, Raz A. Tyrosine-phosphorylated galectin-3 protein is resistant to prostate-specific antigen (PSA) cleavage. J Biol Chem. 2012;287(8):5192-5198. **22232548**.

20. Balan V, Nangia-Makker P, Jung YS, Wang Y, Raz A. Galectin-3: A novel substrate for c-Abl kinase. Biochim Biophys Acta. 2010;1803(10):1198-1205. **20600357**.

21. Li X, Ma Q, Wang J, Liu X, Yang Y, Zhao H, Wang Y, Jin Y, Zeng J, Li J, Song L, Li X, Li P, Qian X, Cao C. c-Abl and Arg tyrosine kinases regulate lysosomal degradation of the oncoprotein Galectin-3. Cell Death Differ. 2010;17(8):1277-1287. **20150913**.

22. Nakajima K, Kho DH, Yanagawa T, Harazono Y, Hogan V, Chen W, Ali-Fehmi R, Mehra R, Raz A. Galectin-3 Cleavage Alters Bone Remodeling: Different Outcomes in Breast and Prostate Cancer Skeletal Metastasis. Cancer Res. 2016;76(6):1391-1402. **26837763**.

23. Joeh E, O'Leary T, Li W, Hawkins R, Hung JR, Parker CG, Huang ML. Mapping glycan-mediated galectin-3 interactions by live cell proximity labeling. Proc Natl Acad Sci U S A. 2020;117(44):27329-27338. **33067390**.

24. Grosveld G, Verwoerd T, van Agthoven T, de Klein A, Ramachandran KL, Heisterkamp N, Stam K, Groffen J. The chronic myelocytic cell line K562 contains a breakpoint in bcr and produces a chimeric bcr/c-abl transcript. Mol Cell Biol. 1986;6(2):607-616. **3023859**.

25. ten Hoeve J, Kaartinen V, Fioretos T, Haataja L, Voncken JW, Heisterkamp N, Groffen J. Cellular interactions of CRKL, and SH2-SH3 adaptor protein. Cancer Res. 1994;54(10):2563-2567. **8168080**.

26. Fei F, Joo EJ, Tarighat SS, Schiffer I, Paz H, Fabbri M, Abdel-Azim H, Groffen J, Heisterkamp N. B-cell precursor acute lymphoblastic leukemia and stromal cells communicate through Galectin-3. Oncotarget. 2015;6(13):11378-11394. **25869099**.

27. Morin NA, Oakes PW, Hyun YM, Lee D, Chin YE, King MR, Springer TA, Shimaoka M, Tang JX, Reichner JS, Kim M. Nonmuscle myosin heavy chain IIA mediates integrin LFA-1 de-adhesion during T lymphocyte migration. J Exp Med. 2008;205(1):195-205. **18195072**.

28. Even-Ram S, Doyle AD, Conti MA, Matsumoto K, Adelstein RS, Yamada KM. Myosin IIA regulates cell motility and actomyosin-microtubule crosstalk. Nat Cell Biol. 2007;9(3):299-309. **17310241**.
